# Circ_HECW2 regulates LPS‐induced apoptosis of chondrocytes via miR‐93 methylation

**DOI:** 10.1002/iid3.453

**Published:** 2021-06-02

**Authors:** Jianwei Zuo, Chen Chen, Xintao Zhang, Jiangyi Wu, Canfeng Li, Shuai Huang, Peiheng He, Qingde Wa, Wentao Zhang

**Affiliations:** ^1^ Department of Sports Medicine Peking University Shenzhen Hospital Shenzhen Guangdong China; ^2^ Department of Anesthesiology Peking University Shenzhen Hospital Shenzhen Guangdong China; ^3^ Department of Orthopedic Surgery The Second Affiliated Hospital of Guangzhou Medical University Guangzhou Guangdong China; ^4^ Department of Joint Surgery The First Affiliated Hospital of Sun Yat‐sen University Guangzhou Guangdong China; ^5^ Department of Orthopedic Surgery The Second Affiliated Hospital of Zunyi Medical University Zun Yi Guizhou China

**Keywords:** apoptosis, chondrocytes, Circ_HECW2, miR‐93, osteoarthritis

## Abstract

**Introduction:**

Circ_HECW2 plays a key role in lipopolysaccharide (LPS)‐induced signal transduction, which is critical in osteoarthritis (OA). Thus, we analyzed the role of Circ_HECW2 in osteoarthritis.

**Methods:**

The expression of Circ_HECW2 and miR‐93 was examined using reverse‐transcription polymerase chain reaction. Cell apoptosis was evaluated using Annexin V‐FITC Apoptosis Detection Kit.

**Results:**

Circ_HECW2 and miR‐93 were inversely correlated, with Circ_HECW2 upregulated and miR‐93 downregulated in OA and LPS‐induced chondrocytes. Circ_HECW2 overexpression inhibited miR‐93 expression and increased methylation of miR‐93 coding gene. Cell apoptosis analysis showed that Circ_HECW2 overexpression increased LPS‐induced chondrocyte apoptosis, while MiR‐93 overexpression reversed the effects of Circ_HECW2 on chondrocyte apoptosis.

**Conclusion:**

In summary, our data revealed that the Circ_HECW2 is highly expressed in OA and might inhibit miR‐93 expression through methylation to affect LPS‐induced chondrocyte apoptosis.

## Graphical abstract

Circ_HECW2 is downregulated in OA and it may downregulate miR‐93 through methylation to promote LPS‐induced apoptosis of chondrocytes

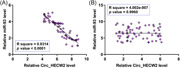

## 1 INTRODUCTION

Osteoarthritis (OA) is the most common type of arthritis, affecting about 10% of males and 13% of females over 60 years of age.^1^ It occurs when the protective cartilage that cushions bone ends wears down over time^2^ and mainly affects knees, hips, hands, and spine to cause disability.^3^ At present, no cure is available for OA.^4^ All currently available treatment approaches, such as acetaminophen, nonsteroidal anti‐inflammatory drugs, and duloxetine, mainly focus on the relief of OA symptoms, primarily pain.^5^, ^6^ Therefore, developing novel and effective approaches is urgently needed for OA treatment.

Molecular targeted therapy is emerging as a novel approach to treat human diseases, including OA.^7^, ^8^ Different from other therapies, targeted therapy treats human diseases mainly by regulating disease‐related gene expression.^7^, ^8^ For instance, Wnt signaling, which plays a critical role in OA, is likely a target for OA treatment.^7^ However, developing novel targets relies on our understanding of the molecular pathogenesis of OA, which is hardly known.^9^, ^10^ Circular RNAs (circRNAs) regulate protein transcription to regulate human diseases,^11^, ^12^ suggesting the potential role of circRNAs as targets for OA treatment.^13^ A recent study shows that Circ_HECW2, derived from exons 12 and 13 of the HECW2 gene, plays a key role in lipopolysaccharide (LPS)‐induced signal transduction,^14^ which is critical in OA pathogenesis.^15^ Our previous microarray analysis revealed that Circ_HECW2 expression is altered in OA, and is inversely correlated with miR‐93.^16^ Studies have shown that miR‐93 regulates apoptosis and inflammation by targeting multiple genes, including interleukin‐1 receptor‐associated kinase 4, signal transducer and activator of transcription 3, B cell lymphoma‐2, and cyclin E1, and may regulate chondrocyte apoptosis to participate in OA.^16^, ^17^, ^18^, ^19^ Yet, no data have shown the potential relationship between Circ_HECW2 and miR‐93. Therefore, more extensive studies are required. Therefore, this study investigated whether and how Circ_HECW2 functions to suppress miR‐93 expression in OA.

## 2 MATERIALS AND METHODS

### 2.1 Patients and controls

No public gene expression database contains the expression of Circ_HECW2 in OA patients, possibly because that Circ_HECW2 is a recently identified circRNA. To analyze the differential expression of Circ_HECW2 in OA, this study enrolled a total of 64 OA patients (60.5 ± 6.3 years, 38 cases of knee OA and 26 cases of hip OA, 40 females and 24 males) and 64 healthy controls (60.4 ± 6.5 years, 40 females and 24 males) at Peking University Shenzhen Hospital between May 2018 and May 2020. To diagnose OA, OA‐related bone changes and damages were revealed by X‐ray. Joint aspiration was also performed on all OA patients to exclude other bone disorders. This study excluded OA patients with preadmission treatment and recurrent OA. The Ethics Committee approved the study before the admission of patients. All patients signed the written informed consent.

### 2.2 Extraction of synovial fluid

All OA patients were subjected to joint aspiration to collect synovial fluid (1.0 ml, affected sites). Synovial fluid (1.0 ml) was also extracted from the corresponding site of healthy controls (38 cases of the knee and 26 cases of the hip) to serve as the control. All synovial fluid samples were stored in liquid nitrogen.

### 2.3 Chondrocytes (OA) and cell culture

Human OA‐affected chondrocytes cells were from Sigma‐Aldrich (402OA‐05A) and cultured in Dulbecco's modified Eagle's medium (11330032; Gibco, Thermo Fisher Scientific) containing 10% fetal bovine serum (10270‐106; Gibco) and 1% penicillin/streptomycin (15140148; Thermo Fisher Scientific) at 37°C under 5% CO_2_. To induce inflammatory injury, chondrocytes were stimulated with LPS (916374, LPS; Sigma‐Aldrich) at various concentrations (0, 1, 5, 10, and 20 μg/ml) for 48 h.

### 2.4 Cell transfections

Circ_HECW2 sequence was designed to insert into pcDNA3.1 plasmid to construct a Circ_HECW2 expression plasmid (pcDNA3.1‐Circ_HECW2). Both pcDNA3.1‐Circ_HECW2 and miR‐93 mimics were purchased from Invitrogen. OA cells were seeded in six‐well plates at a density of 1 × 10^5^ cells per well and incubated overnight to achieve monolayer confluence and transfected with indicated vectors or microRNAs (miRNAs) using Neon Transfection System (MPK5000; Thermo Fisher Scientific) according to the manufacturer's instruction. The cells were transfected for 48 h at 37°C under 5% CO_2_ and then subjected to subsequent assay.

### 2.5 Preparation of RNA samples

Trizol reagent (15596026; Invitrogen) was used to isolate total RNA from synovial fluid and chondrocytes following the manufacturer's protocols. DNase I (18047019; Invitrogen) was added to all RNA samples and incubated at 37°C until an OD260/280 ratio close to 2.0 (indicating pure RNA) was reached. Polyacrylamide gel electrophoresis gel (5%) electrophoresis was performed to analyze RNA integrity. Subsequent assays were performed using high‐quality RNA samples.

### 2.6 Reverse‐transcription polymerase chain reaction analysis

Total RNA was used for reverse transcription (RT) to prepare complementazry DNA (cDNA) samples using SSRT‐IV (18090010, Invitrogen) system. The synthesized cDNA was used as the template to evaluate Circ_HECW2 expression by quantitative reverse‐transcription polymerase chain reaction (RT‐qPCR) analysis using SYBR Green Master Mix (1725270; Bio‐Rad) with 18S rRNA as the internal control. Poly(A) was added to mature miRNAs using all‐in‐one miRNA qRT‐PCR Detection Kit (QP115; GeneCopoeia). The same kit was used to perform miRNA RTs and quantitative PCRs (qPCRs) to measure mature miR‐93 expression levels. The PCR reaction was carried out with Applied Biosystems AB7500 Real‐Time PCR system (Applied Biosystems) at the thermal cycle conditions of 95°C for 10 min and 40 cycles of 95°C for 15 s and 60°C for 1 min. Relative gene expression levels were calculated using the 2‐∆∆Ct method. The primers used for PCR were Circ_HECW2 forward 5′‐CCCACCACTTTGAACGCTAC‐3′ and reverse 5′‐GGCTGTCAATGCGTGCCT‐3′ and miR‐93 forward 5′‐AGGCCCAAAGT GCTGTTCGT‐3′ and reverse 5′‐GTGCAGGGTCCGAGGT‐3′.

### 2.7 Methylation‐specific PCR (MSP)

Genomic DNA was extracted from chondrocytes using the conventional method. EZ DNA Methylation‐Gold Kit (D5006; ZYMO Research) was used to convert DNA samples, followed by performing both methylation‐specific PCRs (MSPs) and routine PCRs to determine the expression of the methylated and unmethylated miR‐93 gene, respectively, using Tag 2X master mix (M0270L, NEB). The primers used in MSP were methylation forward: 5′‐CTGGGGGCTCCAAAGTGCTGTTCG‐3′ and 5′‐CCGGGGGCTCGGGAAGTG CTAG‐3′ and unmethylation forward 5′‐CTGGGGGCTCCAAAGTGCTGTTCG‐3′ and reverse 5′‐CCGGGGGCTCGGGAAGTGCTAG‐3′.

### 2.8 Analysis of cell apoptosis

At 48 h after transfections, chondrocytes cells were seeded into 96‐well plates with 3000 cells in 0.1 ml medium per well. Cells were treated with 10 μg/ml LPS for 48 h in culture media. Then cells were washed with fresh medium, digested by trypsin, and collected by centrifugation. Cell apoptosis was detected using Annexin V‐FITC Apoptosis Detection Kit (15342‐54; nacalai tesque). In brief, cells were resuspended in 195 μl Annexin V‐fluorescein isothiocyanate (FITC) binding solution, followed by the addition of 5 μl Annexin V‐FITC and 10 μl propidium iodide. The mixture was incubated at room temperature in the dark for 30 min. After that, cell apoptosis was analyzed using flow cytometry.

### 2.9 Statistical analysis

Quantitative data were presented as the mean ± *SD* of experimental replicates and processed using GraphPad Prism 6.0. Two and multiple independent groups were compared by unpaired *t* test and analysis of variance Tukey's test, respectively. *p* < .05 was considered statistically significant.

## 3 RESULTS

### 3.1 OA patients exhibited altered expression of Circ_HECW2 and miR‐93

**Figure 1:**
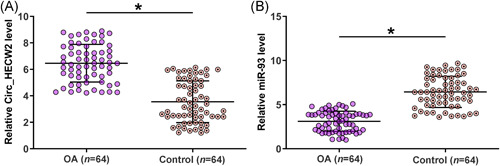
OA patients exhibited altered expression of Circ_HECW2 and miR‐93. Samples of synovial fluid collected from OA patients (*n* = 62) and healthy controls (*n* = 62) were subjected to RT‐qPCR analysis of Circ_HECW2 (A) and miR‐93 (B) expression. OA, osteoarthritis; RT‐qPCR, reverse‐transcription quantitative polymerase chain reaction. *p < .05

To evaluate the expression level of Circ_HECW2 and miR‐93 in OA patient, synovial fluid samples were collected from 64 OA patients and 64 healthy controls. The Total RNAs from synovial fluid samples were extracted, and qPCRs were performed. The results showed that the OA group exhibited increased Circ_HECW2 expression (Figure 1A, *p* < .05) and decreased miR‐93 expression (Figure 1B, *p* < .05) compared with the healthy control group.

### 3.2 LPS treatment altered the expression of Circ_HECW2 and miR‐93

**Figure 2:**
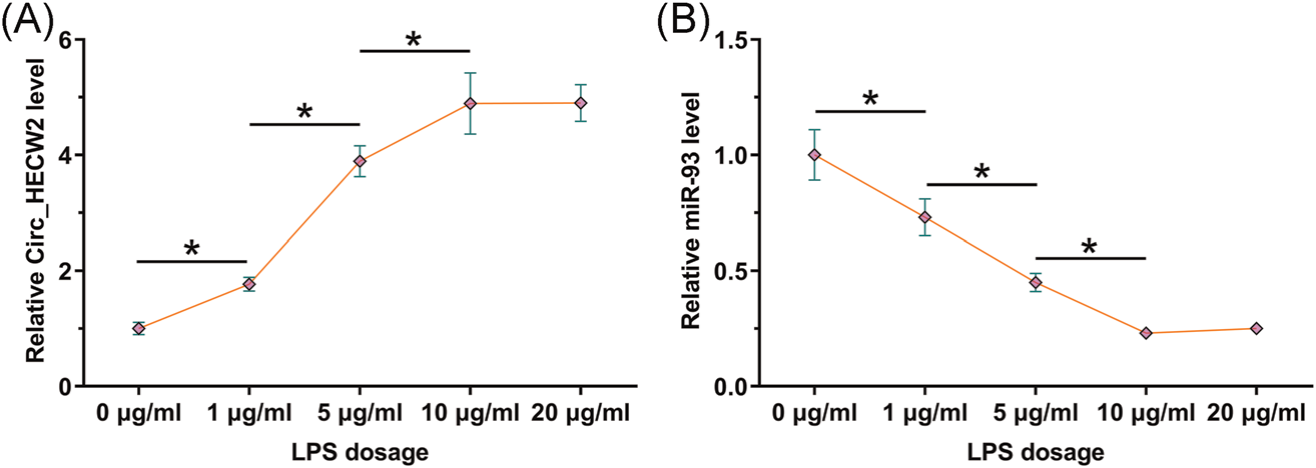
LPS treatment altered the expression of Circ_HECW2 and miR‐93. To analyze the mechanism of OA‐mediated altered expression of Circ_HECW2 and miR‐93, chondrocytes were cultured with LPS at dosages of 0, 1, 5, 10, and 20 μg/ml for 48 h, followed by RT‐qPCR analysis of (A) Circ_HECW2 and (B) miR‐93 expression. LPS, lipopolysaccharide; OA, osteoarthritis; RT‐qPCR, reverse‐transcription quantitative polymerase chain reaction. * *p* < .05

A previous study has shown that LPS‐induced inflammatory injury of chondrocytes can be served as an in vitro OA model. To investigate whether Circ_HECW2 and miR‐93 are involved in LPS‐induced chondrocyte injury, we assessed changes in the expression of Circ_HECW2 and miR‐93 in response to LPS treatment in chondrocytes in vitro. The RT‐qPCR analysis demonstrated that Circ_HECW2 expression was significantly upregulated by LPS exposure in chondrocytes (Figure 2A, *p* < .05). Moreover, LPS exposure also resulted in markedly downregulated miR‐93 expression (Figure 2B, *p* < .05). These data indicated that Circ_HECW2 and miR‐93 expression in OA are altered by LPS, which plays a critical role in OA.

### 3.3 Circ_HECW2 and miR‐93 are closed correlated across OA samples

**Figure 3:**
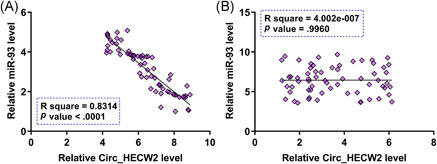
Circ_HECW2 and miR‐93 were closed correlated in OA samples. This figure presents the correlations between Circ_HECW2 and miR‐93 across (A) OA samples and (B) healthy control samples. OA, osteoarthritis

To analyze the correlation between Circ_HECW2 and miR‐93 in OA samples and healthy controls, the correlation analysis was performed. The results showed that Circ_HECW2 and miR‐93 were closed correlated across OA samples (Figure 3A), but not across healthy control samples (Figure 3B). This data suggested that the crosstalk between Circ_HECW2 and miR‐93 is only significant in disease conditions.

### 3.4 Circ_HECW2 overexpression decreases miR‐93 expression through methylation in chondrocytes

**Figure 4:**
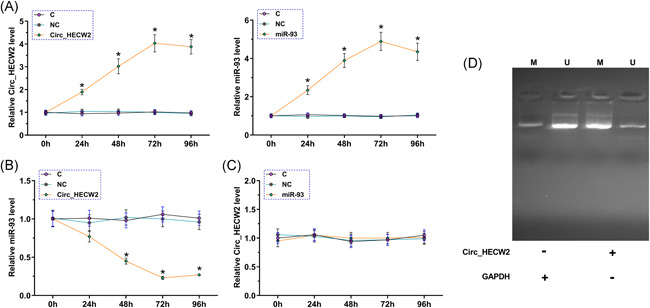
Circ_HECW2 overexpression decreased miR‐93 expression via methylation in chondrocytes. To further test the crosstalk between Circ_HECW2 and miR‐93, chondrocytes were transfected with Circ_HECW2 expression vector or mimic of miR‐93 to overexpress Circ_HECW2 and miR‐93 (A). The role of Circ_HECW2 in miR‐93 expression (B) and the role of miR‐93 in Circ_HECW2 expression (C) were analyzed by RT‐qPCR. (D) MSP was performed to analyze the effects of Circ_HECW2 expression on the methylation of miR‐93 gene. GAPDH, glyceraldehyde 3‐phosphate dehydrogenase; M, methylation; NC, negative control; RT‐qPCR, reverse‐transcription quantitative polymerase chain reaction; U, un‐methylation. *p < .05

To further investigate the crosstalk between Circ_HECW2 and miR‐93, chondrocytes were transfected with Circ_HECW2 expression vector or miR‐93 mimic to overexpress Circ_HECW2 and miR‐93, respectively. The overexpression was monitored and confirmed from 24 to 96 h after transfection (Figure 4A, *p* < .05). In addition, Circ_HECW2 overexpression significantly suppressed miR‐93 expression (Figure 4B, *p* < .05). However, miR‐93 overexpression did not affect Circ_HECW2 expression (Figure 4C, *p* < .05), suggesting that it was the increased Circ_HECW2 level that led to the low miR‐93 level in OA patients and LPS‐induced chondrocytes.

Because DNA methylation is the most common epigenetic modification to regulate gene expression level, we performed MSP analysis to evaluate the methylation level followed by Circ_HECW2 overexpression. The results demonstrated that miR‐93 methylation was upregulated upon Circ_HECW2 overexpression (Figure 4D). Together, these data indicated that Circ_HECW2 overexpression decreased miR‐93 expression via methylation in chondrocytes.

### 3.5 Circ_HECW2 overexpression increases LPS‐induced chondrocyte apoptosis via miR‐93

**Figure 5:**
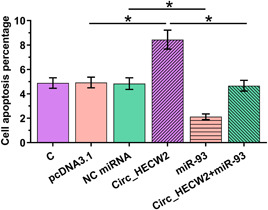
Circ_HECW2 overexpression increased LPS‐induced chondrocyte apoptosis via miR‐93. The transfected chondrocytes were incubated with 10 μg/ml LPS, followed by cell apoptosis assay to analyze the role of Circ_HECW2 and miR‐93 in regulating cell apoptosis. LPS, lipopolysaccharide; miRNA, microRNA; NC, negative control. *p < .05

To explore the biological function of Circ_HECW2 in regulating LPS‐induced chondrocyte injury, we performed overexpression experiments by transfecting Circ_HECW2 expression vector and miR‐93 mimic into LPS‐treated chondrocytes and then analyzed their effects on cell apoptosis. The results showed that Circ_HECW2 overexpression significantly increased apoptosis of LPS‐treated chondrocytes, while miR‐93 overexpression reversed the effect of Circ_HECW2 on cell apoptosis (Figure 5, *p* < .05). This data suggested that Circ_HECW2 overexpression increased apoptosis of LPS‐treated chondrocytes via inhibiting miR‐93 expression.

## 4 DISCUSSION

It has been suggested that Circ_HECW2 and miR‐93 were reversely altered in OA patients. However, the interaction between Circ_HECW2 and miR‐93 and their roles in OA pathogenesis remained unclear. Notably, our findings have revealed an important role of Circ_HECW2 in regulating miR‐93 expression to promote cell apoptosis in OA. Therefore, Circ_HECW2 might severe as a potential target for OA therapy.

Our data showed that Circ_HECW2 was significantly upregulated in OA patients and LPS‐induced OA cell model, consistent with previous findings that Circ_HECW2 is increased in LPS‐induced human brain microvascular endothelial cells^14^ and suggesting the involvement of Circ_HECW2 in OA pathogenesis.

Chondrocytes are the only mature cell components in healthy cartilage, and its apoptosis is one of the major pathogenesis pathways involved in OA.^20^ In this study, we showed that Circ_HECW2 overexpression increased the apoptotic cell number in LPS‐induced chondrocytes, indicating that Circ_HECW2 might play an important role in OA progression.

A previous study demonstrated that miR‐93 inhibited chondrocyte apoptosis and inflammation in osteoarthritis by targeting TLR4/nuclear factor‐κB signaling pathway.^16^ Our data also showed that miR‐93 was downregulated in OA patients and the OA cell model, and it also exhibited a suppression role in chondrocytes apoptosis, consistent with previous publications. We also found that Circ_HECW2 regulates the inhibitory effects of miR‐93 on cell apoptosis via mediating its gene methylation. Therefore, Circ_HECW2 might serve as an upstream inhibitor of miR‐93. Interestingly, Circ_HECW2 was only closely correlated with miR‐93 across OA samples, but not control samples. The underlying mechanism needs to be further identified.

In summary, our findings demonstrated that Circ_HECW2 expression is increased and miR‐93 expression is decreased in OA patients and OA cell models, and their altered expression contributed to the increased cell apoptosis in the OA cell model. We also demonstrated that Circ_HECW2 could serve as an upstream miR‐93 inhibitor by methylating miR‐93 expression gene, and this regulating mechanism could illustrate the promotional role of Circ_HECW2 in chondrocyte apoptosis during OA progression.

## 5 CONCLUSION

In conclusion, Circ_HECW2 is downregulated in OA and may suppress miR‐93 expression via methylation to promote LPS‐induced chondrocyte apoptosis.

## CONFLICT OF INTERESTS

The authors declare that there are no conflict of interests.

## AUTHOR CONTRIBUTIONS

Jianwei Zuo, Chen Chen, and Xintao Zhang contributed to manuscript writing, literature research, experimental work, and data analysis. Jiangyi Wu and Canfeng Li contributed to experimental work, data analysis, and statistical analysis. Shuai Huang, Peiheng He, and Qingde Wa contributed to data review, research design, and statistical analysis. Wentao Zhang contributed to research concept, research design, data review, and manuscript editing. All authors read and approved the final manuscript.

## Data Availability

The data that support the findings of this study are available from the corresponding author upon reasonable request.
